# Safety and Efficacy of Triple Antithrombotic Therapy with Dabigatran versus Vitamin K Antagonist in Atrial Fibrillation Patients: A Pilot Study

**DOI:** 10.1155/2019/5473240

**Published:** 2019-02-13

**Authors:** Vincenzo Russo, Anna Rago, Riccardo Proietti, Emilio Attena, Carmen Rainone, Mario Crisci, Andrea Antonio Papa, Paolo Calabrò, Antonio D'Onofrio, Paolo Golino, Gerardo Nigro

**Affiliations:** ^1^Chair of Cardiology, University of Campania “Luigi Vanvitelli”, Naples, Italy; ^2^Chair of Cardiology, University of Padua, Padua, Italy; ^3^Department of Cardiology, Fatebenefratelli Hospital, Naples, Italy; ^4^Interventional Cardiology Unit, Monaldi Hospital, Naples, Italy; ^5^Departmental Unit of Electrophysiology, Evaluation and Treatment of Arrhythmias, Monaldi Hospital, Naples, Italy

## Abstract

**Background:**

Combination of dual antiplatelet (DAPT) and oral anticoagulation therapy is required to decrease cardioembolic stroke and stent thrombosis risk in patients with atrial fibrillation (AF) and acute coronary syndrome (ACS). We compared the safety and efficacy of dabigatran etexilate with vitamin K antagonist (VKA), in combination with DAPT (aspirin plus clopidogrel) treatment in AF patients who underwent percutaneous coronary intervention (PCI) with stenting for ACS.

**Methods:**

Consecutive nonvalvular AF patients who received twice-daily dabigatran 110 mg (n = 389) or VKA (n = 510) and DAPT were included. Primary endpoints were major bleeding (safety) and the composite of ischemic stroke, systemic embolism, and myocardial infarction (efficacy). The secondary efficacy endpoint was hospitalization for cardiovascular disease.

**Results:**

After propensity score matching, comparative treatment groups comprised 175 dabigatran recipients and 175 VKA recipients. The cumulative incidence of major bleeding was lower in the dabigatran group (2.3%) compared with the VKA group (10.3%) with a hazard ratio (HR) of 4.81 [95% confidence interval (CI) 1.6–14.2,* p* < 0.005]. The cumulative incidence of thromboembolic events with dabigatran was slightly higher (8.0%) than with VKA (6.85%), but not statistically significantly so (0.8, 0.39–1.8;* p* = 0.6). Cumulative incidence of hospitalization for cardiovascular disease was lower with dabigatran (10.3%) compared with VKA (20.6%) treatment (2.2, 1.25–3.8;* p* < 0.006).

**Conclusion:**

Dabigatran at the dose used for stroke prevention appears safer than VKA and maintains a similar efficacy profile, when used with DAPT, in AF patients who have undergone PCI with stenting for ACS.

## 1. Introduction

An overall increase in longevity has resulted in a more frequent association between atrial fibrillation (AF) and percutaneous coronary intervention (PCI) with stenting, in clinical practice. In this regard, approximately 6 to 8% of patients with acute coronary syndrome (ACS) have AF or other conditions for which oral anticoagulation (OAC) is indicated. Furthermore, 20 to 30% of patients with AF experience coexisting ischemic heart disease [1]. Consequently, the combination of dual antiplatelet therapy (DAPT) and OAC is often required: DAPT to prevent cardiovascular events, including stent thromboses, and OAC to reduce the risk of stroke and systemic embolism [2].

Triple antithrombotic therapy, typically comprising a vitamin K antagonist (VKA), aspirin, and clopidogrel, is associated with a high risk of bleeding [3]. Non-vitamin K antagonist oral anticoagulants (NOACs) offer a safe and effective alternative to VKAs for anticoagulation in nonvalvular atrial fibrillation (NVAF) patients [4]. The use of NOACs in association with clopidogrel has resulted in a significantly lower risk of bleeding compared with triple therapy with VKA in two recent studies that included AF patients undergoing PCI [5, 6].

Recent European guidelines [7] recommend a default duration of one to six months of triple therapy in patients at high ischemic risk (e.g., after an ACS). However, the NOACs have not been specifically evaluated in a triple oral antithrombotic therapy regimen for coexisting AF, ACS, and PCI with stenting, so there is no clear worldwide indication in this clinical setting.

Our study aimed to compare the safety and efficacy of dabigatran etexilate with VKA, both in combination with DAPT (comprising aspirin plus clopidogrel), in AF patients who had undergone PCI with stenting for ACS in a clinical practice setting, i.e., outside the arena of a randomized clinical trial.

## 2. Materials and Methods

### 2.1. Database

Data for this study were sourced from the prospectively maintained Atrial Fibrillation Research Database shared by three Italian cardiologic centers in Naples, Italy (Monaldi Hospital, University of Campania “Luigi Vanvitelli”, and Buonconsiglio Hospital), which includes all AF patients followed by these centers. All patients provided written, informed consent before inclusion in the database, and the local institutional review committee approved the study. The database was queried for patients with AF who were prescribed the NOAC dabigatran and VKA anticoagulant therapy and who had a history of coronary angioplasty with stenting for ACS from July 2013 to January 2016.

### 2.2. Patient Population

We consecutively identified 899 patients with nonvalvular atrial fibrillation who underwent coronary angioplasty with stenting for ACS and received OAC treatment (389 dabigatran, 510 VKA) and DAPT (aspirin plus clopidogrel). We excluded patients with a follow-up of less than six months or who were lost to follow-up before six months. To account for baseline differences and potential confounding effects, we used propensity score matching to select a set of dabigatran users and a corresponding set of VKA users.

Follow-up data were obtained through outpatient visits at one, three, and six months. During the follow-up visits, clinical status, treatment adherence (assessed by pill counts), stroke occurrence, transient ischemic attack (TIA), myocardial infarction, major and minor bleeding events, other side effects, and major cardiovascular complications were assessed. Ischemic stroke was defined as a focal neurological deficit lasting for at least 24 hours with no signs of hemorrhage on cerebral imaging and was verified radiologically. The definition of TIA was an acute focal neurological deficit of less than 24-hour duration. Systemic embolism was defined as an acute vascular insufficiency associated with clinical or radiographic evidence of arterial occlusion and not associated with another likely cause. Acute myocardial infarction (MI) was defined according to the European Society of Cardiology third universal definition [8]. Major bleeding was defined as fatal bleeding or symptomatic bleeding in a critical area or organ, or bleeding causing a fall in hemoglobin level of ≥ 2 g/dL or more, or leading to transfusion of two or more units of whole blood or red cells [9].

### 2.3. Endpoints

The primary safety outcome was major bleeding. The primary efficacy outcome was the composite of all events classified as ischemic stroke, systemic embolism, or MI. The secondary efficacy endpoint was hospitalization for cardiovascular disease.

### 2.4. Statistical Analysis

Descriptive statistics of patient characteristics were undertaken; in particular, frequency and percentage were reported for categorical variables, and mean and standard deviation (SD) were used to summarize continuous variables. Bleeding incidence was calculated both as the incidence rate (the ratio between the number of new events occurring during follow-up and the person-time accrued from study participants) every 100 patient-years and as cumulative incidence. T-tests were used to compare continuous variables, and categorical variables were compared using *χ*2 tests. As significant differences existed between patients in the dabigatran and VKA treatment groups in some baseline characteristics, propensity score matching was applied. The estimated propensity score was obtained as the predicted probability of exposure of each patient to dabigatran. Matching was based on the logit of propensity score, using calipers of width 0.2 of the SD of the logit of the propensity score. The absolute standardized differences of the 11 covariates included in the propensity score calculation were compared before and after matching, to assess bias reduction achieved by propensity matching, with a value of < 10% indicating between-group balance. All statistical analyses were performed using IBM SPSS Version 19 (SPSS, Armonk, NY, USA).

## 3. Results

### 3.1. Study Population

Consecutive patients (*n* = 899, mean age 71.8 ± 9.8, 420 female) with AF who underwent coronary angioplasty with stenting for ACS and were treated with OAC (dabigatran etexilate or VKA) and DAPT (aspirin plus clopidogrel) were included in the analysis. Of these, 389 patients were receiving dabigatran 110 mg twice daily, and 510 patients were receiving uninterrupted VKA and had a therapeutic international normalized ratio (INR).

Overall, 18 patients were lost to follow-up: seven in the dabigatran treatment group and 11 in the VKA treatment group.

Propensity score logit matching identified 175 each of twice-daily dabigatran 110 mg and VKA recipients who were comparable in age, gender, body mass index, common comorbidities, CHA_2_DS_2_-VASc score, prevalence of persistent AF, and antiplatelet intake. Because INR would be inherently higher in the VKA group, it was not included in matching.

Baseline characteristics of the study population before and after propensity score matching are summarized in Table 1.

Mean CHA_2_DS_2_-VASc scores (3.5 ± 1.4 versus 3.4 ± 1.6) and HAS-BLED scores (2.03 ± 1.2 versus 2.05 ± 1.4) were similar between dabigatran and VKA groups, respectively, after propensity score adjustment.

The study population included nonvalvular atrial fibrillation patients, 200 with ST elevation ACS and 150 with non-ST elevation ACS, all of whom had undergone PCI with stenting.

### 3.2. Major Bleeding Events

**Figure 1:**
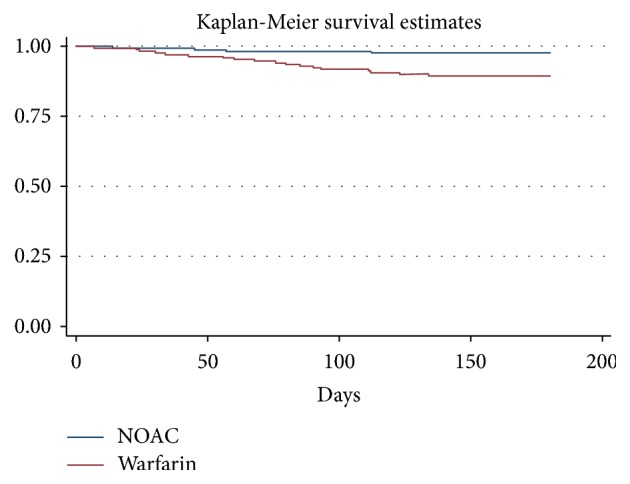
Kaplan-Meier cumulative probability of major bleeding event-free survival in recipients of dabigatran (DAB) and vitamin K antagonist (VKA) treatment.

Twenty-two patients had a major bleeding event. The cumulative incidence of major bleeding was 2.3% (4/175) in dabigatran recipients and 10.3% (18/175) in VKA recipients (HR 4.81, 95% CI 1.6–14.2;* p* < 0.005). The incidence of gastrointestinal bleeding was similar in the two treatment groups [1.1% with dabigatran (2/175) versus 0.6% with VKA (1/175),* p* = 0.3].  Figure 1 shows the Kaplan-Meier cumulative probability of major bleeding event-free survival in dabigatran and VKA treatment groups (log rank* p*-value < 0.005).

### 3.3. Thromboembolic Events

**Figure 2:**
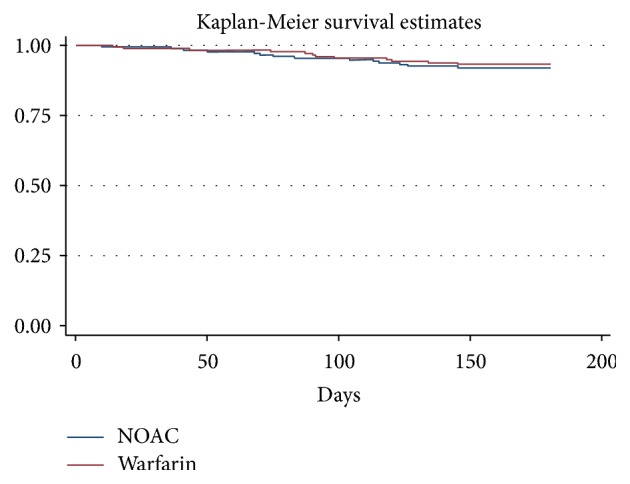
Kaplan-Meier cumulative probability of thrombotic event-free survival in recipients of dabigatran (DAB) and vitamin K antagonist (VKA) treatment.

Twenty-six patients experienced thromboembolic events (ischemic stroke, systemic embolism, or MI) during the follow-up period. The cumulative incidence of thromboembolic events in the dabigatran and VKA groups was 8.0% (14/175) and 6.86% (12/175), respectively [hazard ratio (HR) 0.8, 95% confidence interval (CI) 0.39–1.8;* p* = 0.6].  Figure 2 shows the Kaplan-Meier cumulative probability of thrombotic event-free survival in dabigatran and VKA treatment groups.

There were no statistically significant differences in the cumulative incidence of TIA in the two treatment groups [1.7% in the dabigatran group (3/175) versus 1.1% (2/175) in the VKA group;* p* = 0.3].

### 3.4. Hospitalization

**Figure 3:**
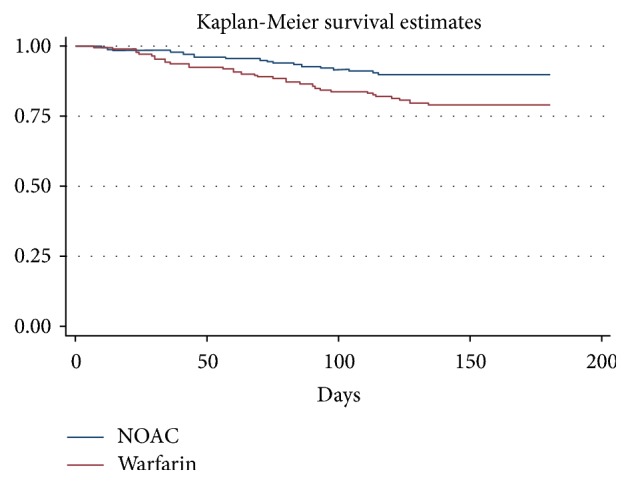
Kaplan-Meier cumulative probability of hospitalization event-free survival in recipients of dabigatran (DAB) and vitamin K antagonist (VKA) treatment.

Fifty-four patients were hospitalized during the follow-up period. The cumulative incidence of hospitalization for cardiovascular disease was 10.3% (18/175) in the dabigatran group and 20.6% (36/175) in the VKA group (HR 2.2, 95% CI 1.25–3.8;* p* < 0.006).  Figure 3 shows the Kaplan-Meier cumulative probability of hospitalization event-free survival in DAB and VKA treatment groups.

### 3.5. Adverse Events and Adherence to Treatment

During follow-up, 14 patients (8%) in the dabigatran treatment group reported adverse events: dyspepsia in seven patients (4%), diarrhea in four patients (2.2%), minor bleeding in two patients (1.1%), and headache in one patient (0.6%). Five patients reported resolution of dyspepsia with concomitant food intake, copious water, proton pump inhibitors, or H2-antagonists. In the VKA group, 15 patients (8.6%) reported adverse events during follow-up: diarrhea in five patients (2.9%), vomiting in four patients (2.2%), nausea in three patients (1.7%), and minor bleeding in three patients (1.7%).

A similar proportion of patients in each treatment group exhibited correct adherence to treatment (92% in dabigatran recipients and 90% in VKA recipients).

## 4. Discussion

Our prospective, observational, propensity score-matched, multicenter cohort study is the first to investigate the safety and efficacy of twice-daily dabigatran etexilate 110 mg versus VKA, in association with DAPT (aspirin plus clopidogrel), in a “real world” clinical practice setting, in AF patients at high ischemic risk who have undergone PCI with stenting for ACS. The safety profile of triple antithrombotic therapy with dabigatran was improved over that of VKA treatment and characterized by a lower cumulative incidence of major bleeding and a lower rate of hospitalization for cardiovascular events. No statistically significant difference in efficacy was found between the two treatments.

Managing AF in patients who have undergone PCI for ACS is challenging. In this clinical setting, OAC reduces the risk of stroke and systemic embolism, whereas DAPT with clopidogrel plus aspirin prevents cardiovascular events, including stent thromboses.

Current guidelines for patients treated with coronary stent implantation recommend triple therapy with OAC plus aspirin and clopidogrel for at least one month, irrespective of the type of stent used. This guidance is increased to a maximum of six-month treatment duration in the presence of high ischemic risk due to acute clinical presentation or anatomical and/or procedural features that outweigh the bleeding risk [4].

Standard triple therapy (VKA, clopidogrel, and aspirin) may minimize the risk of stent thrombosis and ischemic events [3], but it is associated with an increased risk of bleeding. NOACs offer a safe and effective alternative to VKA for anticoagulation in AF [4, 10–16]. However, they have not been specifically tested—at the dose used for stroke prevention—as components of a triple oral antithrombotic therapy regimen for coexisting AF and PCI with stenting; hence, there is no clear worldwide indication for this clinical setting.

Among phase III trials in AF, the RE-LY study, which investigated dabigatran versus the vitamin K antagonist warfarin, was the only trial in which a small proportion of study patients received DAPT with aspirin plus clopidogrel (4.5%;* n* = 812) in addition to a NOAC [17]. In particular, the subanalysis of RE-LY by Hijazi and colleagues [18] suggested that the relative risk of bleeding is similar whether antiplatelets are used with dabigatran or with warfarin. In actuality, the addition of antiplatelets did not affect the benefits of dabigatran over warfarin with regard to efficacy and safety outcomes reported in the main study results. Twice-daily dabigatran 110 mg remained safer than warfarin with regard to major bleeding, and it remained equally effective to warfarin in stroke and systemic embolism risk prevention, regardless of any use of antiplatelet treatment.

The Rocket AF (Rivaroxaban Once Daily Oral Direct Factor Xa Inhibition Compared with Vitamin K Antagonism for Prevention of Stroke and Embolism Trial in Atrial Fibrillation) excluded those patients taking aspirin in combination with thienopyridines within five days prior to randomization; however, 109 patients in VKA group (1.55%) and 143 patients in rivaroxaban group (2.03%) took triple therapy. No significant difference in major bleeding between the two treatment groups was reported, with numerically fewer major bleeds, critical organ bleeds, and fatal bleeds in rivaroxaban-treated patients [19]. No data are available about triple therapy (VKA or NOACs with clopidogrel plus aspirin) from the ARISTOTELE [20] and ENGAGE-AF trials [21].

Most recently, two well-designed trials have found the risk of bleeding with a regimen of reduced dose NOACs plus a P2Y_12_ inhibitor is lower than standard triple therapy with dose-adjusted warfarin [5, 6].

In the PIONEER AF-PCI trial [5], the rates of clinically significant bleeding associated with once-daily rivaroxaban 15 mg (75% of the usual dose) and a P2Y_12_ inhibitor (dual therapy) as well as the rates associated with very low-dose rivaroxaban (2.5 mg twice daily) plus DAPT (triple therapy) were lower than rates experienced with triple therapy with warfarin plus DAPT. However, the PIONEER AF-PCI trial evaluated doses of rivaroxaban that were lower than the dose used for stroke prevention in the ROCKET-AF trial and, furthermore, the PIONEER AF-PCI trial was not powered to detect differences in the prevention of ischemic events. Moreover, although no excess ischemic events were noted in recipients of dual therapy with rivaroxaban 15 mg once daily plus clopidogrel, there was an excess of stroke events with 2.5 mg twice-daily rivaroxaban plus six months' DAPT compared with VKA plus six months' DAPT.

The RE-DUAL PCI trial [6] randomly assigned 2725 patients with AF who had undergone PCI, to receive triple therapy with warfarin plus a P2Y_12_ inhibitor (clopidogrel or ticagrelor) and aspirin (for one to three months); dual therapy with dabigatran at a dose of 110 mg twice daily plus a P2Y_12_ inhibitor; or dual therapy with dabigatran at a dose of 150 mg twice daily plus a P2Y_12_ inhibitor. The rate of International Society on Thrombosis and Haemostasis (ISTH) major or non-major clinically relevant bleeding was lower in the dabigatran 110 mg dual therapy group than in the triple therapy group (HR 0.52, 95% CI 0.42–0.63;* p* < 0.001 for noninferiority) and was also lower in the dabigatran 150 mg dual therapy group than in the corresponding triple therapy group (HR 0.72; 95% CI 0.58–0.88;* p* < 0.001 for noninferiority). There were markedly lower rates of intracranial bleeding in the dabigatran 110 mg and 150 mg groups (0.3% and 0.1%, respectively) compared with the triple therapy group (1.0%). Both dual therapies were noninferior to triple therapy with respect to the composite efficacy endpoint of death, MI, stroke, systemic embolism, or unplanned revascularization. Therefore, the RE-DUAL PCI trial showed dual therapy with dabigatran plus clopidogrel or ticagrelor resulted in a risk of bleeding significantly lower than that of triple therapy. Furthermore, dual therapy with dabigatran was noninferior to triple therapy for ischemic event prevention.

A recent open-label study by Gaubert and colleagues [22] concludes that, in AF patients undergoing PCI for an ACS, replacement of VKA with dabigatran and clopidogrel is associated with an increased thrombotic risk, without a reduction in major bleeding.

This study compared a cohort of 133 consecutive patients receiving dabigatran plus clopidogrel, with an equal number of patients treated with VKA plus clopidogrel (control group). After propensity score adjustment, the cumulative incidence of major adverse cardiovascular events over a 24-month period was higher in dabigatran recipients than in VKA recipients (adjusted HR 2.28, 95% CI 1.46–3.56). Similar rates of major bleeding were found (adjusted HR 1.17, 95% CI 0.46–2.96).

The authors of the recent TALENT-AF (The internAtionaL stENT-Atrial Fibrillation study) [23] find that although more patients with AF who require PCI receive OACs at presentation, the use of triple therapy is not more frequent in “Recent” cohorts of patients, than in “Prior” cohorts. TALENT-AF was a multicenter retrospective registry study in which the authors compared temporal trends between prior (2010–2012) and recent (2013–2015) cohorts of patients with AF requiring PCI and investigated antiplatelet and OAC prescriptions at discharge. Compared with the prior cohort, more patients in the recent cohort received OACs (44.3% versus 56.9%,* p* = 0.01) and NOACs (3.6 versus 27.3%,* p* < 0.01) at baseline, but at discharge, receipt of triple therapy was not different between the cohorts. Clinical presentation with ACS and ensuing potent P2Y12 inhibitor use were associated with reduced odds of a prescription for OAC upon discharge.

Literature analysis identifies an evident lack of data concerning the safety and efficacy of NOACs, at the dose used for stroke prevention, in association with DAPT (aspirin plus clopidogrel) in AF patients who have undergone PCI with stenting for ACS in clinical practice.

### 4.1. Limitations

This was a nonrandomized observational study which compared, after propensity score matching, a small number of AF patients who received twice-daily dabigatran 110 mg or VKA and DAPT. Although we found no statistically significant difference in the rates of the primary efficacy outcome, a lack of statistical power prevents us from drawing firm conclusions about individual event rates.

## 5. Conclusions

Triple antithrombotic therapy with the NOAC dabigatran etexilate 110 mg twice daily in combination with DAPT (aspirin plus clopidogrel) in AF patients who have undergone PCI with stenting for ACS appears safer than triple antithrombotic therapy with VKA and maintains a similar efficacy profile. This therapeutic strategy, which includes dabigatran at the dose tested for stroke prevention and the availability of a specific reversal agent, should be adopted for at least one month and up to six months in this patient population. Further randomized controlled studies are necessary to confirm our preliminary results.

## Figures and Tables

**Table 1:** Study population baseline characteristics before and after propensity matching.

Variable	Before propensity score matching			After propensity score matching		
	DAB	VKA	*P* value	DAB	VKA	*P* value
	(*n *= 389)	(*n *= 510)		(*n *= 175)	(*n *= 175)	
Age (years)	65.1 ± 10.1	75.8 ± 6.5	<0.001	60.2 ± 12.0	62.2 ± 11.5	0.83
Female (%)	42.8	43.1	0.68	43.07	42.09	0.71
BMI (kg/m^2^)	27.8 ± 7.1	27.9 ± 7.1	0.82	27.8 ± 6.1	28.8 ± 6.1	0.78
Hypertension (%)	48.5	59.1	0.001	50.2	49.9	0.57
CHA_2_DS_2_-VASc score	3.3 ± 1.5	4.1 ± 1.4	0.02	3.5 ± 1.4	3.4 ± 1.6	0.55
HAS-BLED score	2.1 ± 1.2	3.1 ±1.1	0.001	2.03 ± 1.2	2.05 ±1.4	0.55
Diabetes mellitus (%)	12	20	0.06	12	11	0.4
Heart failure (%)	19.8	28.2	0.001	20.9	21.2	0.8
Prior stroke/TIA (%)	28.6	38.2	0.001	28.7	27.8	0.6
Prior MI (%)	7.1	13.1	0.02	5.5	6.3	0.6
CrCl (ml/min)	70.2 ± 19.1	61.2 ± 14.5	0.001	70.2 ± 22.1	73.1 ± 20.2	0.7
LAD (mm)	45.3 ± 5.9	46.4 ± 5.8	0.8	46.1 ± 6.3	47.2 ± 5.2	0.8
LAVI (ml/m^2^)	32.1 ± 1.1	34.2 ± 0.9	0.42	32.7 ± 2.2	33.5 ± 1.1	0.7
LVEF (%)	55.3 ± 8.5	45.2 ± 7.1	0.001	55.2 ± 5.2	53.2 ± 4.2	0.8

Values are mean ± SD unless otherwise stated.

*BMI*: body mass index, *CrCl*: creatinine clearance, *DAB:* dabigatran etexilate, *LAD*: left atrial diameter, *LAVI*:indexed left atrial volume, *LVEF*: left ventricle ejection fraction,* MI*: myocardial infarction, *SD*: standard deviation, *TIA*: transient ischemic attack, and *VKA*: vitamin K antagonist.

## Data Availability

The data used to support the findings of this study come from the Arial Fibrillation Research Database shared by three Italian cardiologic centers. Data are available from Dott.Vincenzo Russo (v.p.russo@libero.it) and Dott.ssa Anna Rago (anna_rago@alice.it) for researchers who meet the criteria for access to confidential data.
